# Modifiable Factors Associated with Non-adherence to Antihypertensive or Antihyperlipidemic Drugs Are Dissimilar: a Multicenter Study Among Patients with Diabetes in Indonesia

**DOI:** 10.1007/s11606-020-05809-y

**Published:** 2020-04-16

**Authors:** Sofa D. Alfian, Nurul Annisa, Fajriansyah Fajriansyah, Dyah A. Perwitasari, Rizky Abdulah, Eelko Hak, Petra Denig

**Affiliations:** 1 Unit of PharmacoTherapy, -Epidemiology, & -Economics, Groningen Research Institute of Pharmacy, University of Groningen, Groningen, the Netherlands; 2 Faculty of Pharmacy, Department of Pharmacology and Clinical Pharmacy, Universitas Padjadjaran, Jatinangor, Indonesia; 3 Center of Excellence in Higher Education for Pharmaceutical Care Innovation, Universitas Padjadjaran, Jatinangor, Indonesia; 4 Faculty of Pharmacy, Unit of Clinical Pharmacy and Community, Universitas Mulawarman, Samarinda, Indonesia; 5 Faculty of Pharmacy, Sekolah Tinggi Ilmu Farmasi Makassar, Makassar, Indonesia; 6 Faculty of Pharmacy, Department of Clinical Pharmacy, Universitas Ahmad Dahlan, Yogyakarta, Indonesia; 7 Department of Clinical Pharmacy and Pharmacology, University of Groningen, University Medical Center Groningen, Groningen, the Netherlands

**Keywords:** medication non-adherence, medication beliefs, diabetes mellitus, blood pressure–lowering medication, lipid-lowering medication

## Abstract

**Background:**

To develop targeted and tailored interventions for addressing medication non-adherence, it is important to identify underlying factors.

**Objective:**

To identify factors associated with non-adherence as well as subtypes of non-adherence to antihypertensive or antihyperlipidemic drugs among patients with type 2 diabetes in Indonesia.

**Design:**

An observational multicenter cross-sectional survey.

**Participants:**

Patients with type 2 diabetes using either antihypertensive or antihyperlipidemic drugs in four regions in Indonesia.

**Main Measures:**

Non-adherence and its subtypes of intentional and unintentional non-adherence were assessed using the Medication Adherence Report Scale. Necessity and concern beliefs were assessed with the Beliefs about Medicines Questionnaire. We applied binary and multinomial logistic regression to assess associations of medication beliefs, sociodemographic factors, and clinical-related factors to non-adherence and report odds ratios (OR) with 95% confidence intervals (CI).

**Key Results:**

Of 571 participating patients (response rate 97%), 45.5% and 52.7% were non-adherent to antihypertensive and antihyperlipidemic drugs, respectively. Older age was associated with non-adherence to antihypertensive drugs (60–69 years) (OR, 5.65; 95% CI, 2.68–11.92), while higher necessity beliefs (OR, 0.92; 95% CI, 0.88–0.95) were associated with less non-adherence. Factors associated with non-adherence to antihyperlipidemic drugs were female gender (OR, 1.84; 95% CI, 1.03–3.27) and higher concern beliefs (OR, 1.10; 95% CI, 1.03–1.18), while higher necessity beliefs (OR, 0.89; 95% CI, 0.83–0.96) were associated with less non-adherence.

**Conclusions:**

The main factors associated with non-adherence to antihypertensive and antihyperlipidemic drugs are modifiable. In general, beliefs about the necessity of the drug are important but for antihyperlipidemic drugs concerns are important as well. Healthcare providers should pay attention to identify and address medication beliefs during patient counselling.

**Electronic supplementary material:**

The online version of this article (10.1007/s11606-020-05809-y) contains supplementary material, which is available to authorized users.

## INTRODUCTION

Diabetes is an emerging chronic disease in developing countries, including Indonesia.^1^ The number of patients with diabetes in Indonesia was 10.3 million in 2017, and this number is expected to increase to 16.7 million by 2045.^2^ Patients with diabetes have a higher prevalence rate of cardiovascular disease (CVD) than adults without diabetes,^3^ which is a major cause of comorbidity and mortality.^4^ Hypertension and hyperlipidemia are common in patients with diabetes and contribute significantly to an increased risk of CVD.^5^ Therefore, antihypertensive and antihyperlipidemic co-medication is often necessary in diabetes patients.^5^

Although antihypertensive and antihyperlipidemic drugs are fully covered by health insurance in Indonesia, medication adherence to these drugs is known to be suboptimal,^6^ which may lead to poor health outcomes and increased healthcare costs.^7^ The risk of non-adherence to antihypertensive and antihyperlipidemic drugs is high due to the asymptomatic nature of these diseases, that is, the lack of noticeable efficacy by the patient in everyday life.^8, 9^ Patients with diabetes may have particular problems with their adherence to antihypertensive and antihyperlipidemic co-medication. While much research has been conducted to assess adherence to their antidiabetic drugs and its underlying factors,^10, 11^ there is limited knowledge regarding their adherence to cardiovascular co-medication.

In Indonesia, the guidelines emphasize the importance of addressing medication adherence during patient counselling in the pharmacy,^12^ community health center (CHC),^13^ and hospital.^14^ However, there is no clear evidence of which information or focus is needed to improve medication adherence. Several studies have identified possible factors associated with medication non-adherence among patients with diabetes in developed^15, 16^ and developing countries.^17^ However, these studies have explored largely non-modifiable factors with a weak association between most sociodemographic or drug-related factors and medication non-adherence. In other settings, medication beliefs were found to be one of the important modifiable factors associated with intentional (a conscious decision after balancing the pros and cons of a medication) and unintentional (lack of understanding or forgetfulness) non-adherence.^18–22^ Medication beliefs in general among patients in Asia^23^ and in particular to cardiovascular drugs in Indonesia^21^ were reported low. The Necessity–Concern Framework emphasizes that medication beliefs consist of the necessity of drugs based on beliefs about the positive effects and concerns about the adverse consequences of taking a drug.^24^ A meta-analytic review using this framework including patients with diabetes in developed countries showed that stronger beliefs of necessity and fewer concerns about treatment were associated with higher adherence.^25^

Although results are not fully consistent, it seems that unintentional and intentional non-adherence can differ among the therapeutics groups as reported by patients.^26, 27^ Particularly, differences in concerns may be associated with differences in intentional non-adherence, whereas difference in numbers of drugs needed per indication may be associated with difference in unintentional non-adherence.^26, 27^ To develop a targeted and tailored intervention, insight into the relation between necessity and concern beliefs and non-adherence to antihypertensive and antihyperlipidemic drugs among patients with type 2 diabetes in Indonesia is needed.

The primary objective of this study is to identify factors associated with non-adherence to antihypertensive and antihyperlipidemic drugs among patients with type 2 diabetes in Indonesia with a focus on medication beliefs. The secondary objective is to identify factors associated with different subtypes of non-adherence to antihypertensive and antihyperlipidemic drugs among these patients.

## METHODS

### Study Design, Setting, and Recruitment of Patients

We conducted an observational multicenter cross-sectional survey among patients with type 2 diabetes in four regions in Indonesia (Bandung City, Makassar City, Samarinda City, and Yogyakarta City). In each region, at least five community health centers (CHCs), locally called *puskesmas*, were selected as sampling sites. CHCs are primary healthcare centers at the subdistrict level, with each center staffed with medical doctors, nurses, midwives, and pharmacists. The CHCs were purposively selected based on a sufficient number of diabetes patients with hypertension and/or hyperlipidemia.

We collected data from October 2018 to March 2019 from patients who met the following inclusion criteria: aged over 18 years, with a diagnosis of type 2 diabetes at least 1 year, were prescribed antihypertensive and/or antihyperlipidemic drugs for at least 3 months (prevalent users), and were literate. We excluded patients who had their medication picked up by someone else. The Health Research Ethics Committee of Universitas Padjadjaran approved the study protocol (no. 1137/UN6.KEP/EC/2018).

### Outcomes

Adherence was assessed using the Medication Adherence Report Scale (MARS), which has shown to perform well on a number of psychometric indicators and internal-reliability.^28^ The MARS has been translated and validated to Indonesian and showed to be valid and reliable*.*^29^ The MARS contains one item that reflects unintentional non-adherence (“I forget to take my lipid-lowering medicines”) and four items that largely reflect different forms of intentional non-adherence (e.g., “I alter the dose of my lipid-lowering medicines”).^28^ Patients indicate how often each item applied to them in the last 3 months on a 5-point Likert scale, where 5, “never”; 4, “rarely”; 3, “sometimes”; 2, “often” and 1, “always”.^28^ Non-adherence is defined as a score of 1 to 3 on any of the items, and adherence as a score of 4 or 5 on all items allowing for rarely missing or changing a dose. We defined the subtypes of non-adherence a priori as follows:

1. Unintentional non-adherence includes patients who report to be non-adherent on unintentional adherence (score 1–3 for item 1) but adherent on all intentional non-adherence items (score 4–5 for items 2–5).
2. Intentional non-adherence includes patients who report some form of intentional non-adherence (score 1–3 for at least one of the items 2–5) but adherent on the unintentional non-adherence item (score 4–5 for item 1).
3. In part intentional non-adherence includes patients who report some form of non-adherence on intentional (score 1–3 for at least one of the items 2–5) and on unintentional non-adherence items (score 1–3 for item 1).

### Potential Factors Associated with Non-adherence

Patients’ beliefs were assessed using Beliefs about Medicines Questionnaire (BMQ)-specific.^24^ The Indonesian version of the BMQ-specific showed to be valid (correlation value of each question to the total score > 0.530) and reliable (Cronbach *α* coefficient of 0.835 and 0.811 for necessity and concern beliefs, respectively) (unpublished manuscript). The BMQ-specific contains five items about necessity beliefs (e.g., “My health at present depends on my lipid-lowering medicines”), five items about concern beliefs (e.g., “I sometimes worry about becoming too dependent on my lipid-lowering medicines”), and one item about side effects (e.g., “My lipid-lowering medicines gives me unpleasant side effects”). Patients indicate how often each item applied to them in the last 3 months on a 5-point Likert scale ranging from “strongly disagree” to “strongly agree” with an overall range from 5 (low necessity, low concern) to 25 (high necessity, high concern). We calculated the necessity–concern differential score by subtracting the scores of the concerns scale from the necessity scale (range − 20 to 20). A positive differential score indicates stronger beliefs in the necessity, while a negative score indicates stronger concern.^24^ The item about experienced side effects was included because of its expected additional role in non-adherence.^30, 31^

Sociodemographic factors included as non-modifiable factors were age at the completion of the questionnaire, gender, highest level of education completed (no formal education/elementary school, junior high school, senior high school, or university), and type of health insurance. Type of health insurance was classified as those whose insurance premium was paid by the government (BPJS-PBI), those whose insurance premium was paid by the patients themselves (BPJS-Non PBI), or those without health insurance. Clinical factors included as non-modifiable factors were obtained from medical records: time since diagnosis of diabetes, hypertension, and/or hyperlipidemia (years) and the most recent systolic blood pressure (SBP), diastolic blood pressure (DBP), and total cholesterol level in the past 3 months.

### Data Collection

The pharmacist on duty at the CHCs screened the patients’ eligibility. Once a patient was deemed eligible, the pharmacist informed the researcher or research assistant to approach the patient and explain the study, and ask to sign informed consent. Consenting patients were asked to report the name of their antihypertensive or antihyperlipidemic drugs and subsequently filled in the MARS and BMQ-specific questionnaire. If patients used both antihypertensive and antihyperlipidemic drugs, the MARS and BMQ-specific questionnaires were administered for each therapeutic group. Patients were asked to complete the questionnaire independently. However, in some cases, elderly patients were allowed to complete the questionnaire verbally. Research assistants collected all other data from the medical records using a predefined data collection form. For those with incomplete or unavailable medical records, diagnostic data were obtained using patients printed record from the private laboratory they had visited.

### Sample Size Calculation

A previous small-scale study showed that non-adherence rates among Indonesian patients with diabetes ranged from 50 to 65% using the MARS questionnaire.^32^ In studies elsewhere, similar and lower non-adherence rates have been found, also using the MARS questionnaire.^26, 33^ Therefore, a minimum sample size of 180 patients per therapeutic drug group was required based on the formula for prediction models with a binary outcome,^34^ when including maximum of 9 possible independent variables in the multivariate analysis and assuming a proportion of non-adherence of 50%. With an expected distribution of 2:1 between patients receiving antihypertensive drugs and antihyperlipidemic drugs, 540 patients need to be recruited in the most conservative scenario of no overlap in the use of both therapeutic groups.

### Data Analysis

Analyses were conducted per therapeutic group. When a patient used both antihypertensive and antihyperlipidemic drugs, they were included for both therapeutic groups. Clinical factors related to hypertension or hyperlipidemia, such as duration of hypertension and/or hyperlipidemia and the most recent SBP, DBP, or total cholesterol level, were included only for the related therapeutic group. Descriptive statistics were used to summarize the patient characteristics. Pearson *χ*^2^ tests, Mann–Whitney tests, or Kruskal–Wallis tests were used to assess univariate associations of patient characteristics with outcomes. Since there were few missing data regarding the MARS and BMQ, we conducted complete-case analyses. However, information about the number of medications and comorbidities could not be obtained for all patients due to incompleteness of medical records. The potential factors found to be associated with the outcomes at a significance level of *p* < 0.25 in univariate analyses (Tables 2 and 3) were included in the initial multivariate models. Two regression models were built for both therapeutic groups. Due to collinearity, the necessity–concern differential score was analyzed in a separate model not including the necessity and concern beliefs. Models with a higher *R*-squared value were then selected. In the first model, binary logistic regression with being adherent or non-adherent as the outcome was conducted to obtain odds ratio (OR) with a 95% confidence interval (CI) with manual backward elimination. In the second model, multinomial logistic regression with being adherent, unintentional non-adherent, intentional non-adherent, and in part intentional non-adherent as the outcomes was conducted to obtain OR and 95% CI. All statistical analyses were carried out using SPSS software (version 25.0; IBM, Armonk, NY, USA).

## RESULTS

### Baseline Characteristics

A total of 571 diabetes patients who were prescribed antihypertensive drugs (492 patients) and/or antihyperlipidemic drugs (245 patients) participated in this study (response rate of 97.1%) from Bandung City (6 CHCs; 133 patients), Makassar City (3 CHCs; 67 patients), Samarinda City (5 CHCs; 162 patients), and Yogyakarta City (18 CHCs; 209 patients). The mean values of MARS scores for those who were prescribed antihypertensive and antihyperlipidemic drugs were 22.2 and 22.1, respectively (Table 1). Less than half of the patients were male and most of patients were aged between 60 and 69 years and graduated from senior high school (Table 1). Patients included in the analyses with antihypertensive drugs had shorter diabetes duration than those in antihyperlipidemic drug analysis. More than half of the patients who were prescribed antihyperlipidemic drugs also received antihypertensive drugs, while one-third of those who were prescribed antihypertensive drugs received antihyperlipidemic drugs (Table 1). The median scores of necessity beliefs and concern beliefs were 15.0 (range 12.0–18.0) and 16.0 (range 12.0–18.0) to antihypertensive drugs, and 14.0 (range 12.0–17.0) and 16.0 (range 13.0–19.0) to antihyperlipidemic drugs, respectively.

**Table 1:** **Patient Characteristics per Therapeutic Group**

Characteristic	Antihypertensive drugs (*N* = 492)	Antihyperlipidemic drugs (*N* = 245)
Gender (%)		
Male	181 (36.8)	72 (29.5)
Missing	-	1 (0.4)
Age in years (%)		
≤ 49	57 (11.6)	24 (9.8)
50–59	162 (32.9)	84 (34.3)
60–69	211 (42.9)	120 (49.0)
≥ 70	60 (12.2)	15 (6.1)
Missing	2 (0.4)	2 (0.8)
Type of insurance (%)		
BPJS-PBI	76 (15.4)	40 (16.3)
BPJS-non PBI	349 (70.9)	143 (58.4)
Without insurance	14 (2.8)	13 (5.3)
Missing	53 (10.8)	49 (20.0)
Last education level (%)		
No formal education/ elementary school	92 (18.7)	41 (16.7)
Junior high school	77 (15.7)	32 (13.1)
Senior high school	226 (45.9)	115 (46.9)
University	91 (18.5)	54 (22.0)
Missing	6 (1.2)	3 (1.2)
Time from diagnosis, mean (SD), years		
Diabetes	4.7 (4.4)	4.9 (4.3)
Missing	75 (15.2)	49 (20.1)
Hypertension	4.4 (4.3)	-
Missing	14 (2.8)	-
Hyperlipidemia	-	3.2 (3.2)
Missing	-	34 (13.9)
Clinical data, mean (SD**)**		
SBP (mmHg)	136.7 (13.9)	-
Missing	9 (1.8)	-
DBP (mmHg)	83.4 (8.1)	-
Missing	9 (1.8)	-
Total cholesterol level (mmol/L)	-	223.5 (50.2)
Missing	-	88 (35.9)
Specific co-medication		
Antihyperlipidemic drug	166 (33.7)	-
Antihypertensive drug	-	166 (67.8)
Medication beliefs, median (IQR)		
BMQ-necessity	15.0 (12.0–18.0)	14.0 (12.0–17.0)
Missing	1 (0.2)	1
BMQ-concern	16.0 (12.0–18.0)	16.0 (13.0–19.0)
Missing	-	1
BMQ-side effects	2.0 (1.0–2.0)	2.0 (1.0–2.0)
Missing	1 (0.2)	1
Necessity–concern differential	− 1.0 (– 3.0 to 3.0)	− 1.0 (− 4.0 to 3.0)
Missing	1 (0.2)	1
MARS score, mean (SD)	22.2 (2.9)	22.1 (2.9)
Missing	-	2 (0.8)

*SD* standard deviation, *IQR* interquartile range, *BMQ* Beliefs about Medicines Questionnaire, *MARS* Medication Adherence Report Scale, *BPJS-PBI* insurance premium was paid by the government, *BPJS-Non PBI* insurance premium was paid by the patients themselves

Around half of patients were non-adherent to antihypertensive and to antihyperlipidemic drugs (45.5% and 52.7%, respectively) (Table 2). Patients were further classified as unintentional (14.4%, mean score 22.3), intentional (13.2%, mean score 20.4), and in part intentional (17.9%, mean score 18.3) non-adherent to antihypertensive drugs, and as unintentional (18.1%, mean score 22.6), intentional (6.6%, mean score 21.4), and in part intentional (28.0%, mean score 18.6) non-adherent to antihyperlipidemic drugs (Table 3).

**Table 2:** **Univariate Associations with Non-adherent to Antihypertensive and/or Antihyperlipidemic Drugs**

Characteristic	Antihypertensive drugs (*N* = 492)			Antihyperlipidemic drugs (*N* = 245)		
	Adherent	Non-adherent	*p* value	Adherent	Non-adherent	*p* value
*N* (%)	268 (54.5)	224 (45.5)		115 (47.3)	128 (52.7)	
MARS score, mean (SD)	23.9 (1.5)	20.2 (2.9)		24.0 (1.6)	20.3 (2.6)	
Male gender (%)	105 (39.2)	76 (33.9)	0.229*^,‡^	41 (35.7)	31 (24.4)	0.056*^,‡^
Age in years (%)			0.000*^,‡^			0.010*^,‡^
≤ 49	47 (17.6)	10 (4.5)		15 (13.2)	9 (7.1)	
50–59	105 (39.3)	57 (25.6)		48 (42.1)	34 (26.8)	
60–69	86 (32.2)	125 (56.1)		45 (39.5)	75 (59.1)	
≥ 70	29 (10.9)	31 (13.9)		6 (5.3)	9 (7.1)	
Type of insurance (%)			0.766*			0.018*^,‡^
BPJS-PBI	47 (18.4)	29 (15.8)		18 (17.0)	22 (24.4)	
BPJS-Non PBI	200 (78.4)	149 (81.0)		85 (80.2)	58 (64.4)	
Without insurance	8 (3.1)	6 (3.3)		3 (2.8)	10 (11.1)	
Last education level (%)			0.000*^,‡^			0.028*^,‡^
Elementary school	41 (15.4)	51 (23.4)		13 (11.3)	28 (22.4)	
Junior high school	33 (12.4)	44 (20.0)		18 (15.7)	14 (11.2)	
Senior high school	146 (54.9)	80 (36.4)		63 (54.8)	51 (40.8)	
University	46 (17.3)	45 (20.5)		21 (18.3)	32 (25.6)	
Time since diagnosis, mean (SD), years						
Diabetes	4.3 (3.7)	5.1 (5.0)	0.328^†^	4.7 (3.6)	5.0 (4.6)	0.493^†^
Hypertension	4.2 (3.8)	4.8 (4.8)	0.258^†^	-	-	
Hyperlipidemia				3.4 (2.5)	3.0 (3.7)	0.001^†,‡^
Clinical data, mean (SD)						
SBP (mmHg)	136.4 (12.6)	137.1 (15.4)	0.556^†^	-	-	-
DBP (mmHg)	83.5 (6.9)	83.2 (9.3)	0.835^†^	-	-	-
Total cholesterol level (mmol/L)	-	-	-	233.0 (55.4)	217.8 (46.1)	0.112^†,‡^
Specific co-medication						
Antihyperlipidemic drug	97 (36.2)	69 (30.8)	0.208*^,‡^	-	-	-
Antihypertensive drug	-	-	-	92 (80.0)	74 (57.8)	0.000*^,‡^
Medication beliefs, median (IQR)						
BMQ-necessity	15.0 (13.0–18.0)	13.0 (10.0–17.0)	0.000^†,‡^	15.0 (13.0–18.0)	14.0 (11.3–16.0)	0.023^†,‡^
BMQ-concern	16.0 (13.0–18.0)	15.0 (10.0–18.0)	0.005^†,‡^	16.0 (12.0–18.0)	17.0 (13.0–20.0)	0.044^†,‡^
BMQ-side effects	2.0 (1.0–2.0)	2.0 (1.0–2.0)	0.083^†,‡^	2.0 (1.0–2.0)	2.0 (1.0–2.0)	0.325^†^
Necessity–concern differential	0 (− 3.0 to 4.0)	− 1.0 (− 4.0 to 3.0)	0.036^†,‡^	0 (− 2.0 to 4.0)	− 2.0 (− 5.0 to 0)	0.000^†,‡^

*Pearson *χ*^2^ test

†Mann–Whitney test

‡Included in initial multivariate model

*MARS* Medication Adherence Report Scale, *SD* standard deviation, *BPJS-PBI* insurance premium was paid by the government, *BPJS-Non PBI* insurance premium was paid by the patients themselves, *SBP* systolic blood pressure, *DBP* diastolic blood pressure, *IQR* interquartile range, *BMQ* Beliefs about Medicines Questionnaire

**Table 3:** **Univariate Associations with Different Subtypes of Non-adherent to Antihypertensive and/or Antihyperlipidemic Drugs**

Characteristic	Antihypertensive drugs (*N* = 492)					Antihyperlipidemic drugs (*N* = 245)				
	Adherent	Unintentional non-adherent	Intentional non-adherent	In part intentional non-adherent	*p* value	Adherent	Unintentional non-adherent	Intentional non-adherent	In part intentional non-adherent	*p* value
*N* (%)	268 (54.5)	71 (14.4)	65 (13.2)	88 (17.9)		115 (47.3)	44 (18.1)	16 (6.6)	68 (28.0)	
MARS score, mean, (SD)	23.9 (1.5)	22.3 (0.8)	20.4 (2.6)	18.3 (3.0)		24.0 (1.6)	22.6 (0.5)	21.4 (1.7)	18.6 (2.4)	
Male gender (%)	105 (39.2)	23 (32.4)	18 (27.7)	35 (39.8)	0.273*	41 (35.7)	12 (27.3)	2 (12.5)	17 (25.4)	0.176*^,‡^
Age in years (%)					0.000*^,‡^					0.033*^,‡^
≤ 49	47 (17.6)	4 (5.6)	1 (1.5)	5 (5.7)		15 (13.2)	3 (6.8)	0	6 (9.0)	
50–59	105 (39.3)	17 (23.9)	18 (27.7)	22 (25.3)		48 (42.1)	7 (15.9)	4 (25.0)	23 (34.3)	
60–69	86 (32.2)	42 (59.2)	34 (52.3)	49 (56.3)		45 (39.5)	30 (68.2)	10 (62.5)	35 (52.2)	
≥ 70	29 (10.9)	8 (11.3)	12 (18.5)	11 (12.6)		6 (5.3)	4 (9.1)	2 (12.5)	3 (4.5)	
Type of insurance (%)					0.662*					0.796*
BPJS-PBI	47 (18.4)	7 (12.5)	7 (14.6)	15 (18.8)		18 (17.0)	9 (31.0)	0	13 (27.7)	
BPJS-Non PBI	200 (78.4)	47 (83.9)	41 (85.4)	61 (76.3)		85 (80.2)	17 (58.6)	13 (92.9)	28 (59.6)	
Without insurance	8 (3.1)	2 (3.6)	0	4 (5.0)		3 (2.8)	3 (10.3)	1 (7.1)	6 (12.8)	
Last education level (%)					0.003*^,‡^					0.101*^,‡^
Elementary school	41 (15.4)	21 (30.0)	10 (15.4)	20 (23.5)		13 (11.3)	8 (18.2)	3 (18.8)	17 (26.2)	
Junior high school	33 (12.4)	10 (14.3)	14 (21.5)	20 (23.5)		18 (15.7)	6 (13.6)	1 (6.3)	7 (10.8)	
Senior high school	146 (54.9)	23 (32.9)	27 (41.5)	30 (35.3)		63 (54.8)	16 (36.4)	10 (62.5)	25 (38.5)	
University	46 (17.3)	16 (22.9)	14 (21.5)	15 (17.6)		21 (18.3)	14 (31.8)	2 (12.5)	16 (24.6)	
Time since diagnosis, mean (SD), years										
Diabetes	4.3 (3.7)	4.9 (5.0)	4.6 (4.2)	5.6 (5.4)	0.582^†^	4.7 (3.6)	4.6 (4.8)	7.6 (6.6)	4.5 (3.7)	0.206^†,‡^
Hypertension	4.2 (3.8)	4.7 (5.1)	4.2 (4.1)	5.2 (5.0)	0.258^†^					
Hyperlipidemia						3.4 (2.5)	3.1 (4.0)	3.3 (4.1)	2.9 (3.5)	0.009^†,‡^
Clinical data, mean (SD)										
SBP (mmHg)	136.4 (12.6)	136.5 (13.1)	136.7 (16.6)	137.9 (16.2)	0.919^†^	-	-	-	-	-
DBP (mmHg)	83.5 (6.9)	82.7 (9.4)	83.0 (7.0)	83.8 (10.6)	0.684^†^	-	-	-	-	-
Total cholesterol level (mmol/L)	-	-	-	-	-	233.0 (55.4)	214.0 (52.1)	223.9 (51.7)	218.2 (40.2)	0.009^†,‡^
Specific co-medication										
Antihyperlipidemic drug	97 (36.2)	23 (32.4)	19 (29.2)	27 (30.7)	0.628*	-	-	-	-	-
Antihypertensive drug	-	-	-	-	-	92 (80.0)	25 (56.8)	10 (62.5)	39 (57.4)	0.003*,^‡^
Medication beliefs, median (IQR)										
BMQ-necessity	15.0 (13.0–18.0)	13.0 (11.0–16.0)	14.0 (9.5–18.0)	12.5 (10.0–17.0)	0.000^†,‡^	15.0 (13.0–18.0)	14.0 (11.0–15.0)	14.0 (12.0–15.0)	15.0 (12.0–17.8)	0.067^†,‡^
BMQ-concern	16.0 (13.0–18.0)	14.0 (10.0–17.0)	14.0 (10.5–17.0)	16.0 (12.0–18.8)	0.005^†,‡^	16.0 (12.0–18.0)	16.0 (13.0–19.0)	17.0 (13.0–19.8)	17.0 (10.5–20.0)	0.239^†,‡^
BMQ-side effects	2.0 (1.0–2.0)	2.0 (1.0–2.0)	2.0 (1.0–2.0)	1.0 (1.0–2.0)	0.354^†^	2.0 (1.0–2.0)	2.0 (1.0–2.8)	2.0 (1.0–2.0)	2.0 (1.0–2.0)	0.666^†^
Necessity–concern differential	0 (− 3.0 to 4.0)	− 1.0 (− 4.0 to 4.0)	0 (− 4.0 to 4.5)	− 2.0 (− 5.0 to 0.8)	0.019^†,‡^	0 (− 2.0 to 4.0)	− 3.0 (− 4.0 to − 1.0)	− 2.0 (− 6.0 to 0.0)	− 0.5 (− 5.0 to 3.0)	0.001^†,‡^

*Pearson χ^2^ test

†Kruskal–Wallis test

‡Included in initial multivariate model

*MARS* Medication Adherence Report Scale, *SD* standard deviation, *BPJS-PBI* insurance premium was paid by the government, *BPJS-Non PBI* insurance premium was paid by the patients themselves, *SBP* systolic blood pressure, *DBP* diastolic blood pressure, *IQR* interquartile range, *BMQ* Beliefs about Medicines Questionnaire

### Factors Associated with Non-adherence to Antihypertensive Drugs

From the univariate analyses, gender, age, last education level, specific co-medication, necessity beliefs, concern beliefs, side effects, and necessity–concern differential were selected as potential factors associated with non-adherence (Table 2). In the multivariate model, older age (60–69 years) (OR, 5.65; 95% CI, 2.68–11.92) was associated with non-adherence to antihypertensive drugs, while higher necessity beliefs (OR, 0.92; 95% CI, 0.88–0.95) was associated with less non-adherence (Table 4). The goodness-of-fit *p* value of this model was .351 with an *R*-squared value of 15.2%. The model including the necessity–concern differential had a lower *R*-squared value of 12.7% (Table S1 in Supplementary data). Similar patterns were seen for the subtypes of non-adherence. Patients with higher necessity beliefs were less likely to be unintentional (OR, 0.91; 95% CI, 0.86–0.97), intentional (OR, 0.93; 95% CI, 0.87–0.98), and in part intentional non-adherent (OR, 0.92; 95% CI, 0.87–0.97) (Table 4). Patients aged 60–69 years showed the highest odds of being unintentional, intentional, and in part intentional non-adherent (Table 4).

**Table 4:** **Factors Associated with Non-adherence and Different Subtypes of Non-adherence to Antihypertensive Drugs in Patients with Diabetes**

Factors	Odds ratios* (95% CI)			
	Non-adherence^†^ (*n* = 224)	Unintentional non-adherence^‡^ (*n* = 71)	Intentional non-adherence^‡^ (*n* = 65)	In part intentional non-adherence^‡^ (*n* = 88)
Age in years (*n*)				
≤ 49	Reference	Reference	Reference	Reference
50–59	2.37 (1.11–5.07)	1.72 (0.54–5.45)	7.52 (0.97–58.16)	1.82 (0.65–5.13)
60–69	5.65 (2.68–11.92)	4.17 (1.38–12.61)	15.59 (2.05–118.49)	4.59 (1.69–12.51)
≥ 70	4.14 (1.74–9.82)	2.24 (0.60–8.36)	15.81 (1.93–129.84)	3.10 (0.96–10.08)
BMQ-necessity	0.92 (0.88–0.95)	0.93 (0.87–0.98)	0.93 (0.88–0.99)	0.91 (0.86–0.96)
BMQ-concerns	NA	0.94 (0.87–1.01)	0.98 (0.91–1.06)	1.04 (0.97–1.11)

*Final multivariate model

†Assessed by binary logistic regression with goodness-of-fit *p* value of non-adherence model, 0.351; *R*-squared, 15.2%

‡Assessed by multinomial logistic regression with adherent as a reference outcome group. Overall fit of the different subtypes of non-adherence model: likelihood ratio chi-squared test, *p* < 0.05; pseudo *R*-squared, 14.7%

### Factors Associated with Non-adherence to Antihyperlipidemic Drugs

From the univariate analyses, gender, age, type of insurance, last education level, duration of hyperlipidemia, total cholesterol level, specific co-medication, necessity beliefs, concern beliefs, and necessity–concern differential were selected as potential factors associated with non-adherence (Table 2). In the multivariate model, significant factors associated with non-adherence to antihyperlipidemic drugs were higher concern beliefs (OR, 1.10; 95% CI, 1.03–1.18) and female gender (OR, 1.84; 95% CI, 1.03–3.27), while higher necessity beliefs (OR, 0.89; 95% CI, 0.83–0.96) was associated with less non-adherence (Table 5). The goodness-of-fit *p* value of this model was .716 with an *R*-squared value of 9.1%. The model including the necessity–concern differential had a lower *R*-squared value of 6.6% (Table S2 in Supplementary data). Regarding different subtypes of non-adherence, 1-year increase on the duration of diabetes was associated with an increase in the likelihood of intentional non-adherence (OR, 1.16; 95% CI, 1.04–1.30). Furthermore, patients with higher necessity beliefs were less likely to be unintentional non-adherent (OR, 0.89; 95% CI, 0.80–0.98), and patients with higher concern beliefs were more likely to be both unintentional (OR, 1.11; 95% CI, 1.01–1.22) and intentional (OR, 1.19; 95% CI, 1.03–1.37) non-adherent (Table 5).

**Table 5:** **Factors Associated with Non-adherence and Different Subtypes of Non-adherence to Antihyperlipidemic Drugs in Patients with Diabetes**

Factors	Odds ratios* (95% CI)			
	Non-adherence^†^ (*n* = 128)	Unintentional non-adherence^‡^ (*n* = 44)	Intentional non-adherence^‡^ (*n* = 16)	In part intentional non-adherence^‡^ (*n* = 68)
Female gender	1.84 (1.03–3.27)	NA	NA	NA
Duration of diabetes (years)	NA	1.02 (0.93–1.13)	1.16 (1.04–1.30)	1.01 (0.92–1.10)
BMQ-necessity	0.89 (0.83–0.96)	0.89 (0.80–0.98)	0.88 (0.76–1.02)	0.95 (0.87–1.04)
BMQ-concern	1.10 (1.03–1.18)	1.11 (1.01–1.22)	1.19 (1.03–1.37)	1.08 (0.99–1.17)

*Final multivariate model

†Assessed by binary logistic regression with goodness-of-fit *p* value of non-adherence model, 0.716; *R*-squared, 9.1%

‡Assessed by multinomial logistic regression with adherent as a reference outcome group. Overall fit of the different subtypes of non-adherence model: likelihood ratio chi-squared test, *p* < 0.05; pseudo *R*-squared, 9.3%

## DISCUSSION

Around half of patients with type 2 diabetes being prescribed antihypertensive or antihyperlipidemic drugs in our study were non-adherent to this medication. Older age was associated with non-adherence to antihypertensive drugs, while higher necessity beliefs were associated with less non-adherence. Factors associated with non-adherence to antihyperlipidemic drugs were higher concern beliefs and female gender, while higher necessity beliefs were associated with less non-adherence. In addition, longer duration of diabetes was associated with intentional non-adherence to antihyperlipidemic drugs.

We observed that patients with higher necessity beliefs were less likely to be non-adherent to antihypertensive as well as to antihyperlipidemic drugs. There were not much differences in factors associated with the subtypes of non-adherence to antihypertensive drugs indicating that necessity beliefs are relevant for both unintentional and intentional non-adherence. In patients with chronic diseases, perceived need may affect both unintentional and intentional non-adherence, such that the unintentional behavior may mediate intentional non-adherence.^19^ A previous study among the general population in Indonesia showed that the reason for intentional non-adherence to antihypertensive drugs was a lack of necessity beliefs, in such patients with asymptomatic conditions like hypertension often perceive the need for medications to a lesser extent.^35^ Our study showed that this is also the case in patients with type 2 diabetes.

Furthermore, higher concern beliefs were associated with non-adherence to antihyperlipidemic drugs. Similar results were observed in subtypes of non-adherence to antihyperlipidemic drugs, indicating that concern beliefs are relevant for both unintentional and intentional non-adherence. In contrast, concern beliefs were not associated with non-adherence to antihypertensive drugs. Previous studies showed that concern beliefs may be important for adherence to antihypertension, antihyperlipidemia, antidiabetic, asthma, osteoporosis, or depression.^19^ It could be that concerns regarding antihyperlipidemic drugs are fueled by statin denialism or skepticism but it is not known to what extend this is shared in low- and middle-income countries. It is also possible that more recent initiation of antihyperlipidemic drugs played a role. New users to chronic medication more often become intentionally non-adherent due to side effects and concerns about medication.^36^ In our study, we did not know the time of initiation but the duration of hyperlipidemia was on average shorter than the duration of hypertension. Furthermore, differences in the prevalence of polypharmacy may have been relevant. Polypharmacy is a known factor associated with lower adherence in general.^37, 38^ Two-third of diabetes patients who were prescribed antihyperlipidemic drugs were also using antihypertensive drugs concurrently, whereas only one-third of those who were prescribed antihypertensive drugs used antihyperlipidemic drugs.

We found that older patients (> 49 years) were more likely to be non-adherent to antihypertensive drugs compared with younger patients, while no such association was found with antihyperlipidemic drugs. It is possible that older patients in our study may have experienced more side effects of antihypertensive drugs compared to antihyperlipidemic drugs. On the other hand, patients with longer duration of diabetes were more likely to be intentional non-adherent to antihyperlipidemic drugs, while no association was observed with unintentional non-adherence nor with any type of non-adherence to antihypertensive drugs. It could be that this difference is influenced by different perceptions regarding the long-term benefits of these drugs in patients with more comorbidities.^39^ Finally, females were more likely to be non-adherent to antihyperlipidemic drugs but this association was lost in the analysis of subtypes of non-adherence. No association was found between gender and non-adherence to antihypertensive drugs. This is in line with conflicting results regarding gender in previous studies.^30, 40, 41^ In general, it seems that gender is not a very meaningful factor associated with non-adherence.

Overall, most sociodemographic and clinical factors were not associated with non-adherence to antihypertensive or antihyperlipidemic drugs in our study. Sociodemographic factors, such as education level, may be too general to predict an individual’s medication taking behavior. This is in line with a previous study that showed understanding the importance of treatment is more important than the level of education.^42^ Using specific co-medication was not associated with non-adherence either to antihypertensive or antihyperlipidemic drugs. This finding suggests that type of specific co-medication may be not a relevant factor associated with non-adherence. Surprisingly, side effects were not associated with non-adherence in our study. One could expect, however, that patients who experienced serious or frequent side effects already stopped taking these drugs and therefore were not included in our study.

The strength of this study is that we studied non-adherence to antihypertensive and antihyperlipidemic drugs in the same population, allowing us to study similarities and differences in associated factors. In addition, by using the MARS questionnaire, we were able to make a distinction between unintentional, intentional, and in part intentional non-adherence to identify specific factors associated with different subtypes of non-adherence. Furthermore, the high response rate observed in this study makes the results generalizable for the Indonesian population visiting CHCs for type 2 diabetes. This study was conducted in different CHCs in different regions of Indonesia which strengthens the generalizability of the study.

Some limitations need to be mentioned. Underestimating of non-adherence may have occurred because self-reporting was used for its assessment. Pill counts or pharmacy databases would allow for an objective assessment of adherence but such measures are not widely available in Indonesia. Moreover, pill counts and pharmacy databases cannot provide information regarding the types of non-adherence (intentional or unintentional). The MARS scale has been shown to correlate well with other indirect methods, including pill counts among patients with hypertension and refill rates (using medication possession ratio) among patients with stroke.^43, 44^ Furthermore, the subtype analyses sometimes included small numbers leading to wide confidence intervals and loss of power. Due to the cross-sectional design, no causal inferences can be made regarding the temporal association between medication beliefs and non-adherence. The overall association of our models was relatively low, indicating that there are other unmeasured factors that may influence non-adherence, for example, the total number of medications used or having other comorbidities.

## CONCLUSIONS

Medication beliefs were a potentially modifiable factor associated with non-adherence to antihypertensive as well as to antihyperlipidemic drugs. In general, beliefs about the necessity of the drug are important but for antihyperlipidemic drugs concerns about the drug are important as well. Healthcare providers should pay attention to identify and address medication beliefs during patient counselling.

## Electronic Supplementary Material

ESM 1 (DOCX 40.1 kb)

## References

[1] Islam SMS, Purnat TD, Phuong NTA, Mwingira U, Schacht K, Fröschl G (2014). Non-Communicable Diseases (NCDs) in developing countries: a symposium report. Global Health..

[2] International Diabetes Federation (IDF). IDF Diabetes Atlas. http://www.diabetesatlas.org/resources/2017-atlas.html. Published 2017.

[3] Sarwar N, Gao P, Kondapally Seshasai SR (2010). Diabetes mellitus, fasting blood glucose concentration, and risk of vascular disease: A collaborative meta-analysis of 102 prospective studies. Lancet..

[4] **Einarson TR**, **Acs A**, **Ludwig C**, **Panton UH**. Prevalence of cardiovascular disease in type 2 diabetes: A systematic literature review of scientific evidence from across the world in 2007–2017. *Cardiovasc Diabetol*. 2018;17(1). doi:10.1186/s12933-018-0728-6 10.1186/s12933-018-0728-6 PMC5994068 29884191

[5] American Diabetes Association. 10. Cardiovascular Disease and Risk Management: *Standards of Medical Care in Diabetes—2019*. *Diabetes Care*. 2019;42(Supplement 1):S103-S123. doi:10.2337/dc19-S010 10.2337/dc19-S010 30559236

[6] **Akrom A**, **Anggitasari W**. Adherence and quality of life on diabetic patients with hypertension at bantul public hospital in yogyakarta special region, indonesia. *Int J Public Heal Sci*. 2018;8(1). doi:10.11591/ijphs.v8i1.15240

[7] **Khan R**, **Socha-Dietrich K**. Investing in medication adherence improves health outcomes and health system efficiency: adherence to medicines for diabetes, hypertension, and hyperlipidaemia. *OECD Heal Work Pap*. 2018;(105). 10.1787/8178962c-en

[8] Ho PM, Bryson CL, Rumsfeld JS (2009). Medication adherence: its importance in cardiovascular outcomes. Circulation..

[9] Osterberg L, Blaschke T (2005). Adherence to medication. N Engl J Med..

[10] Alfian S, Sukandar H, Lestari K, Abdulah R (2016). Medication adherence contributes to an improved quality of life in type 2 diabetes mellitus patients : a cross-sectional study. Diabetes Ther..

[11] Krass I, Schieback P, Dhippayom T (2015). Adherence to diabetes medication: A systematic review. Diabet Med..

[12] **Indonesia KKR**. *Peraturan Menteri Kesehatan Republik Indonesia Nomor 73 Tahun 2016 Tentang Standar Pelayanan Kefarmasian Di Apotek*. Jakarta; 2016.

[13] **Indonesia KKR**. *Peraturan Menteri Kesehatan Republik Indonesia Nomor 74 Tahun 2016 Tentang Standar Pelayanan Kefarmasian Di Puskesmas*. Jakarta; 2016.

[14] **Indonesia KKR**. *Peraturan Menteri Kesehatan Republik Indonesia Nomor 72 Tahun 2016 Tentang Standar Pelayanan Kefarmasian Di Rumah Sakit*. Jakarta; 2016.

[15] Kirkman MS, Rowan-Martin MT, Levin R (2015). Determinants of adherence to diabetes medications: findings from a large pharmacy claims database. Diabetes Care..

[16] Tunceli K, Zhao C, Davies MJ (2015). Factors associated with adherence to oral antihyperglycemic monotherapy in patients with type 2 diabetes. Patient Prefer Adherence..

[17] **Alfian SD**, **Sukandar H**, **Arisanti N**, **Abdulah R**. Complementary and alternative medicine use decreases adherence to prescribed medication in diabetes patients. *Ann Trop Med Public Heal*. 2016;9(3). 10.4103/1755-6783.179108

[18] Schüz B, Marx C, Wurm S (2011). Medication beliefs predict medication adherence in older adults with multiple illnesses. J Psychosom Res..

[19] **Gadkari AS**, **Mchorney CA**. Unintentional non-adherence to chronic prescription medications: How unintentional is it really? *BMC Health Serv Res*. 2012;12. doi:10.1186/1472-6963-12-98. 10.1186/1472-6963-12-98 PMC3375198 22510235

[20] Mann DM, Ponieman D, Leventhal H, Halm EA (2009). Predictors of adherence to diabetes medications: the role of disease and medication beliefs. J Behav Med..

[21] Karuniawati H, Ikawati Z, Gofir A (2017). Adherence to secondary stroke prevention therapies in ischemic stroke patients at teaching hospital in Central Java Indonesia. Asian J Pharm Clin Res.

[22] Phatak HM, Thomas J (2006). Relationships between beliefs about medications and use of prescribed chronic medications. Ann Pharmacother..

[23] Horne R, Graupner L, Frost S, Weinman J, Wright SM, Hankins M (2004). Medicine in a multi-cultural society: the effect of cultural background on beliefs about medications. Soc Sci Med..

[24] Horne R, Weinman J, Hankins M (1999). The beliefs about medicines questionnaire: The development and evaluation of a new method for assessing the cognitive representation of medication. Psychol Heal..

[25] **Horne R**, **Chapman SCE**, **Parham R**, **Freemantle N**, **Forbes A**, **Cooper V**. Understanding patients’ adherence-related beliefs about medicines prescribed for long-term conditions: A meta-analytic review of the Necessity-Concerns Framework. *PLoS One*. 2013;8(12). 10.1371/journal.pone.0080633 10.1371/journal.pone.0080633 PMC3846635 24312488

[26] de Vries ST, Keers JC, Visser R (2014). Medication beliefs, treatment complexity, and non-adherence to different drug classes in patients with type 2 diabetes. J Psychosom Res..

[27] Stack RJ, Bundy CE, Eliott RA, New JP, Gibson M, Noyce PR (2010). Intentional and unintentional non-adherence in community dwelling people with type 2 diabetes: The effect of varying numbers of medicines. Br J Diabetes Vasc Dis..

[28] **Chan AHY**, **Horne R**, **Hankins M**, **Chisari C**. The Medication Adherence Report Scale (MARS-5): a measurement tool for eliciting patients’ reports of non-adherence. *Br J Clin Pharmacol*. 2019. doi:10.1111/bcp.14193 10.1111/bcp.14193 PMC7319010 31823381

[29] Alfian R, Putra AMP (2017). Uji validitas dan reliabilitas kuesioner medication adherence report scale (MARS) terhadap pasien diabetes mellitus [in Bahasa Indonesia]. J Ilm Ibnu Sina..

[30] van der Laan DM, Elders PJM, Boons CCLM, Beckeringh JJ, Nijpels G, Hugtenburg JG (2017). Factors associated with antihypertensive medication non-adherence: a systematic review. J Hum Hypertens..

[31] **Birtcher K.** When compliance is an issue—how to enhance statin adherence and address adverse effects. *Curr Atheroscler Rep*. 2015;17(1). doi:10.1007/s11883-014-0471-8 10.1007/s11883-014-0471-8 25410047

[32] Adikusuma W, Perwitasari DA, Supadmi W (2014). Evaluasi kepatuhan pasien diabetes melitus tipe 2 di Rumah Sakit Umum PKU Muhammadiyah Bantul, Yogyakarta [in Bahasa Indonesia]. Media Farm..

[33] Lee CS, Tan JHM, Sankari U, Koh YLE, Tan NC (2017). Assessing oral medication adherence among patients with type 2 diabetes mellitus treated with polytherapy in a developed Asian community: a cross-sectional study. BMJ Open..

[34] Peduzzi P, Concato J, Kemper E, Holford TR, Feinstein AR (1996). A simulation study of the number of events per variable in logistic regression analysis. J Clin Epidemiol..

[35] **Rahmawati R**, **Bajorek B**. Factors affecting self-reported medication adherence and hypertension knowledge: A cross-sectional study in rural villages, Yogyakarta Province, Indonesia. *Chronic Illn*. 2018;14(3):212–227. doi:10.1177/1742395317739092 10.1177/1742395317739092 29119817

[36] Barber N, Parsons J, Clifford S, Darracott R, Horne R (2004). Patients’ problems with new medication for chronic conditions. Qual Saf Health Care..

[37] Cramer JA (2004). A systematic review of adherence with medications for diabetes. Diabetes Care..

[38] Gellad WF, Grenard JL, Marcum ZA (2011). A systematic review of barriers to medication adherence in the elderly: looking beyond cost and regimen complexity. Am J Geriatr Pharmacother..

[39] Gibson DS, Nathan AG, Quinn MT, Laiteerapong N (2018). Patient expectations of hypertension and diabetes medication: Excessive focus on short-term benefits. SAGE Open Med..

[40] Donnelly LA, Doney ASF, Morris AD, Palmer CNA, Donnan PT (2008). Long-term adherence to statin treatment in diabetes. Diabet Med..

[41] Mann DM, Woodward M, Muntner P, Falzon L, Kronish I (2010). Predictors of nonadherence to statins: a systematic review and meta-analysis. Ann Pharmacother..

[42] Krueger KP, Berger BA, Felkey B (2005). Medication adherence and persistence: A comprehensive review. Adv Ther..

[43] **Lin C-Y**, **Huang-Tz Ou**, **Nikoobakht M**, **et al.** Validation of the 5-Item medication adherence report scale in older stroke patients in Iran. *J Cardiovasc Nurs*. 2018;00(0). 10.1097/JCN.0000000000000488 10.1097/JCN.0000000000000488 29649015

[44] **Tedla YG**, **Bautista LE**. Factors associated with false-positive self-reported adherence to antihypertensive drugs. *J Hum Hypertens*. 2017;31(5). 10.1038/jhh.2016.80 10.1038/jhh.2016.80 PMC6062205 27853149

