# Supplementary material for: Modifiable Factors Associated with Non-adherence to Antihypertensive or Antihyperlipidemic Drugs Are Dissimilar: a Multicenter Study Among Patients with Diabetes in Indonesia
Source: J Gen Intern Med. 2020 Apr 16;35(10):2897–906. doi: 10.1007/s11606-020-05809-y (PMC7573041; doi:10.1007/s11606-020-05809-y)
Supplement: Supplementary file 1 — (DOCX 40.1 kb) [file 11606_2020_5809_MOESM1_ESM.docx]

**Supplementary data**

**Table S1 Factors associated with non-adherence to antihypertensive drugs in patients (N = 492) with diabetes by binary logistic regression.**

| **Factors** ^*^ | **Odds Ratios (95% CI)**^†^ |
| --- | --- |
| Age in years (n) |  |
| ≤ 49 (57) | Reference |
| 50–59 (162) | 2.50 (1.17–5.33) |
| 60–69 (211) | 6.68 (3.20–14.00) |
| ≥ 70 (60) | 5.13 (2.19–12.04) |
| Necessity–concern differential^‡^ | 0.96 (0.92–0.99) |

Note:

^*^ Goodness-of-fit p-value: 0.181, R-squared: 12.7%

^†^ Final multivariate model.

^‡^ Necessity–concern differential instead of BMQ-necessity and BMQ-concern beliefs.

**Table S2 Factors associated with non-adherence to antihyperlipidemic drugs in patients (N = 245) with diabetes by binary logistic regression.**

| **Factor**^*^ | **Odds Ratios (95% CI)**^†^ |
| --- | --- |
| Necessity–concern differential^‡^ | 0.91 (0.86–0.96) |

Note:

^*^ Goodness-of-fit p-value: 0.001, R-squared: 6.6%

^†^ Final multivariate model.

^‡^ Necessity–concern differential instead of BMQ-necessity and BMQ-concern beliefs.
